# Supplementary material for: Prevalence of active trachoma and associated factors among children 1–9 years old at Arsi Negele Town, West Arsi Zone, Oromia Regional State, Southern Ethiopia
Source: PLoS One. 2022 Oct 7;17(10):e0273808. doi: 10.1371/journal.pone.0273808 (PMC9543983; doi:10.1371/journal.pone.0273808)
Supplement: Attachment — Submitted filename: TRACHOMA A PUBLIC DISODER REVIEW.docx [file pone.0273808.s002.docx]

TRACHOMA – A PUBLIC HEALTH ISSUE

To live a healthy lifestyle we need to follow a proper healthy diet and follow proper sanitation and stay hygiene, otherwise we will face a variety of diseases and allergies which could lead to severe medical issues and have to follow a lot of medications. So, the paper which im reviewing today is a topic related to the improper sanitation issues which cause a blindness disorder called Trachoma, which is commonly found in the areas with improper hygiene and sanitation mostly such kind of disorder is found in African countries like Nigeria ,Sudan and many other sub Saharan African nations.

Trachoma is a kind of disease which causes conjunctival infection in eye and eventually cause infection in retina and hence cause blindness. The infection spreads easily from person to person with nasal and ocular secretions, and along with sharing of towels, hankerchief etc. This type of infection spreads due to lack of proper sanitation and hygiene. It is mostly seen in the African children aged 1-9 years of age. A survey was considered among the children of aged 1-9 living in dirty quarters and dirty conditions like no proper sanitation and the areas without good water facilities and also the children living in neat and clean hygienic environment. A team has been formed to conduct the survey and they collected the data of their survey and made a list of the population and checked the impact of the infection by using statistical analysis by the help of IBM IT industry. The list was made by focusing on the age, sex , ethnicity like indirect factors and direct factors like water and cooking conditions which play a vital role to check the spreading and virulence of the pathogen.

After collecting the data they found that the infection spread faster to the children staying in unhygienic conditions rather those children staying in hygienic environment. The unhygienic environment gives exposures to children towards conditions like faeces in their house compound, using of dirty towels, flies moving around the faces of the children, improper washing of face without using soap and without using hand washes sanitizer etc, lack of water supply and using of dirty contaminated water of rivers and ponds. The infection is basically a bacterial infection caused by Chlamydia trachomatis a bacteria which causes conjunctival infection initially and eventually cause retinal damage. The symptoms of Trachoma is irritation in eyes, watery eyes, redness, improper vision etc.

According to WHO it says 5% prevalence to tackle Trachoma but in reality after doing a legitimate survey they found that the prevalence of active Trachoma in children 1-9 was about 21.91%. The Bacteria is present on the contaminated surfaces and unhygienic locations years and onto which flies used to fly around and these flies carries the pathogen and contaminate the food, household things, skin surface etc and hence cause the spreading of the infection. The study also found that the children with the filthy faces were three times more likely to have active Trachoma.

So, according to this paper it says by taking proper sanitation and maintaining well sanitized environment and good living conditions can eradicate this microbial pathogen which is really a good report made by the people involved in it. I really appreciate their work to make us aware regarding this serious infection, But as a reviewer of this paper I would suggest if they would have mention the virulence of this particular pathogen causing conjunctival infection and its complete pathogenic period in the child’s body in a molecular biology and biochemistry context like how it eventually leads to retinal damage then it would be much more interesting and informative. And also I got a question regarding how the bacteria isn’t get damaged after entering the eye because as we known that eye contains lysosomal enzyme which has the capability to destroy bacterial cell wall. These information should be present in this paper. However it’s really a good survey……….
