# Supplementary material for: Prevalence of active trachoma and associated factors among children 1–9 years old at Arsi Negele Town, West Arsi Zone, Oromia Regional State, Southern Ethiopia
Source: PLoS One. 2022 Oct 7;17(10):e0273808. doi: 10.1371/journal.pone.0273808 (PMC9543983; doi:10.1371/journal.pone.0273808)
Supplement: Attachment — Submitted filename: Response to reviewers.docx [file pone.0273808.s003.docx]

We would like to thank the editors and reviewers for their constructive comments.

We have presented our responses for each concern as follows;

Journal requirements

1.  Please ensure that your manuscript meets PLOS ONE's style requirements, including those for file naming.

Response: the manuscript and the file name are in line with on plos one format.

2. We note that you have referenced (ie. Bewick et al. [5]) which has currently not yet been accepted for publication. Please remove this from your References and amend this to state in the body of your manuscript: (ie “Bewick et al. [Unpublished]”) as detailed online in our guide for authors

Response: we have corrected it as per the comment given.

Reviewer 1

… But as a reviewer of this paper I would suggest if they would have mention the virulence of this particular pathogen causing conjunctival infection and its complete pathogenic period in the child’s body in a molecular biology and biochemistry context like how it eventually leads to retinal damage then it would be much more interesting and informative. And also I got a question regarding how the bacteria isn’t get damaged after entering the eye because as we known that eye contains lysosomal enzyme which has the capability to destroy bacterial cell wall. This information should be present in this paper. However it’s really a good survey……….

Response: thank you for your concern. However, this study is not an experimental study that investigates molecular biology and bio-chemistry. Besides, the aim of this was not to answer how a bacterium is not damaged after entering eye. This is a simple survey study aimed to assess the prevalence of Trachoma infection and associated factors

Reviewer 2

 1. The study was conducted between 1-9 years old children but more numbers (71.9%) were present between 1-5 years than 6-9 years (28.1%) in Arsi Negele Town also in line 353 they have motioned that is higher than the WHO recommendation for trachoma elimination. The age associated factor for this trachoma infection is lacking in the discussion part and only the result is provided. The authors can modify it accordingly.

Response: Thank you for your thorough review. However, in the discussion section, we did not address these variables. Because, in the multivariate analysis there was no association between respondents' age and the prevalence of active trachoma.

2. Since it’s a medical terminology the abbreviation for TF (Trachoma Folliculitis) can be produced in the text itself particularly in the abstract and I think no need to give separate list for this especially in abbreviation. Sudden confusion may rise in the abstract while reading the terminology TF.

Response: we have corrected it as per the comment given.

3. The regional wise information regard the hygienic practices of the localized study area was given as nice but relating with the hygienic practices of environmental (regional) factors associated with trachoma infection is not scientifically discussed in this manuscript.

Response: the authors strongly believe that the factors associated with Trachoma infection have been discussed thoroughly and scientifically. Therefore, we kindly request the editor to re-check this again.

4. However the clean and unclean faces, flies over the faces were observed in this study as the significant factors associated with the Trachoma infection, but the condition caused blindness and which stage has more risk towards it is essentially need to illustrate in this study.

Response: The WHO staging that causes blindness is Trachoma trichiasis which is very common in individual over 35 years old. Therefore it is not common in children. That is why it is not illustrated in this study.

5. Lacking of potential figures in this manuscript.

Response: we have included one additional figure

6. Over all the manuscript is looks like a report of the clinical study but the scientific illustration from the observation is not fulfilled the criteria and does not give a representation of scientific research temper.
Hence I request the authors to complete and rework with the above suggestions under major revision of the manuscript.

Response: it is known that in this study data were collected through face to face interview and observation and this has been described in detail in the method section. The findings are also presented in the result section (line 273-281) and (line 309-313). Therefore, we kindly request the reviewer to recheck the concern again.
